# Coarctation of the aorta in adults: what is the best treatment? Case report and literature review

**Published:** 2011-05-25

**Authors:** R Jurcut, AM Daraban, A Lorber, D Deleanu, MS Amzulescu, C Zara, BA Popescu, C Ginghina

**Affiliations:** * ‘Carol Davila’ University of Medicine and Pharmacy, BucharestRomania; ** Department of Cardiology, ‘Prof. dr. C.C. Iliescu’ Institute for Emergency in Cardiovascular Diseases, BucharestRomania; *** Department of Pediatric Cardiology, RAMBAM Health Care CampusIsrael; **** Department of Cardiology, Brasov Military Hospital, BrasovIsrael

**Keywords:** aortic coarctation, balloon angioplasty, covered stent, surgery

## Abstract

Coarctation of the aorta is a congenital cardiac malformation that can go undiagnosed until old age with only hypertension as a marker of its presence because clinical signs can be subtle and overlooked if a complete physical exam is not performed.

We report the case of a 45 year–old women, diagnosed with severe coarctation of the aorta just distal to the left subclavian artery, with poststenotic dilatation of the descending aorta and difficult control of blood pressure values. The patient was successfully treated interventionally, by balloon angioplasty with deployment of a covered stent.

We review here the different methods employed for the treatment of coarctation of the aorta in adults, including surgical or percutaneous balloon angioplasty with or without stent placement, underlying their complications and the factors that influence the choice of the best coarctation repair method.

The incidence of coarctation of the aorta is 4 in 10.000 live births, accounting for 5–8% of the children with congenital heart defects [1]

Aortic coarctation presenting during adult life most frequently represents cases of re–coarctation, following previous transcatheter or surgical therapy, or missed cases of native coarctation.

Without correction, the mean life expectancy of patients with aortic coarctation is 35 years and 90% of those patients die before reaching the age of 50 years. Systemic hypertension, accelerated coronary heart disease, stroke, aortic dissection, and congestive heart failure are common complications in patients who have not had surgery or who are operated on in later childhood or adult life.

There are different methods employed for the treatment of CoA in adults, including surgical or percutaneous balloon angioplasty with or without stent placement, and medical therapy.

## Case presentation

We report the case of a 45–year–old woman, diagnosed with arterial hypertension at the age of 39, with difficult control of blood pressure values under treatment with beta–blocker, ACE and diuretics, who was referred to us for evaluation. She had no history of cardiac problems or family history of hypertension.

The clinical examination showed a normal body development, a blood pressure of 145/80 mmHg in the right arm and 135/80 mmHg in the left arm, large pulsations in the suprasternal notch, normal heart sounds, 60 bpm. A systolic murmur was audible at the parasternal right and left area and at the paravertebral interscapular area bilaterally. The radial pulses were palpable but no pulse was found at the palpation of the femoral arteries or distal to that level. 

**Figure 1:**
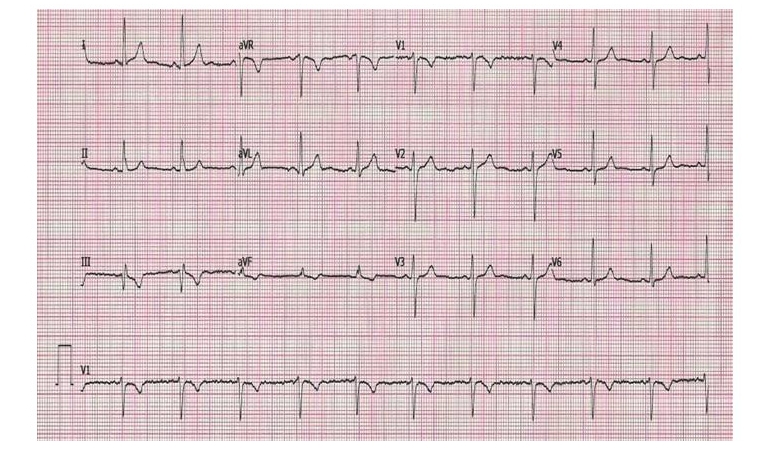
Electrocardiogram. Sinus rythm, 62 bpm, without criteria for LVH.

The electrocardiogram (Figure 1) showed sinus rhythm, QRS axis at +10°, with no criteria for ventricular hypertrophy. 

**Figure 2:**
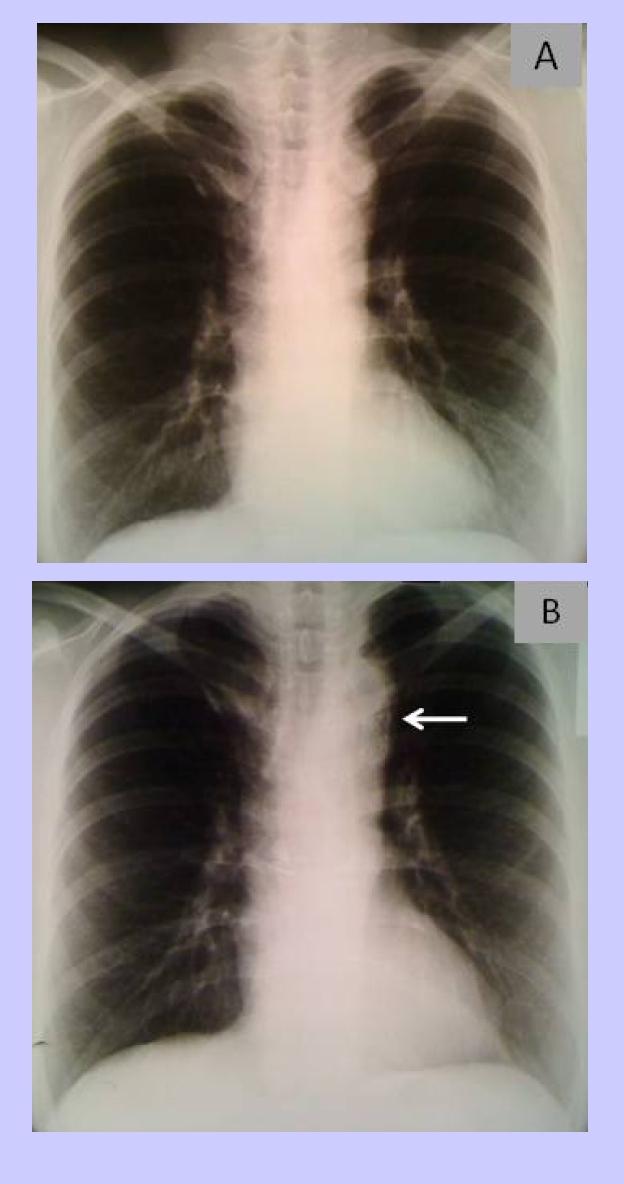
Chest radiography. A Before stent placement and B  After stent placement (see arrow for stent position)

The chest X–ray shows a normal cardiothoracic index, visible azygos vein fissure, a high positioned aortic arch with a discreet ‘3’ sign (biarcuat appearance of the aortic arch) and no rib notching. (Figure 2)

**Figure 3:**
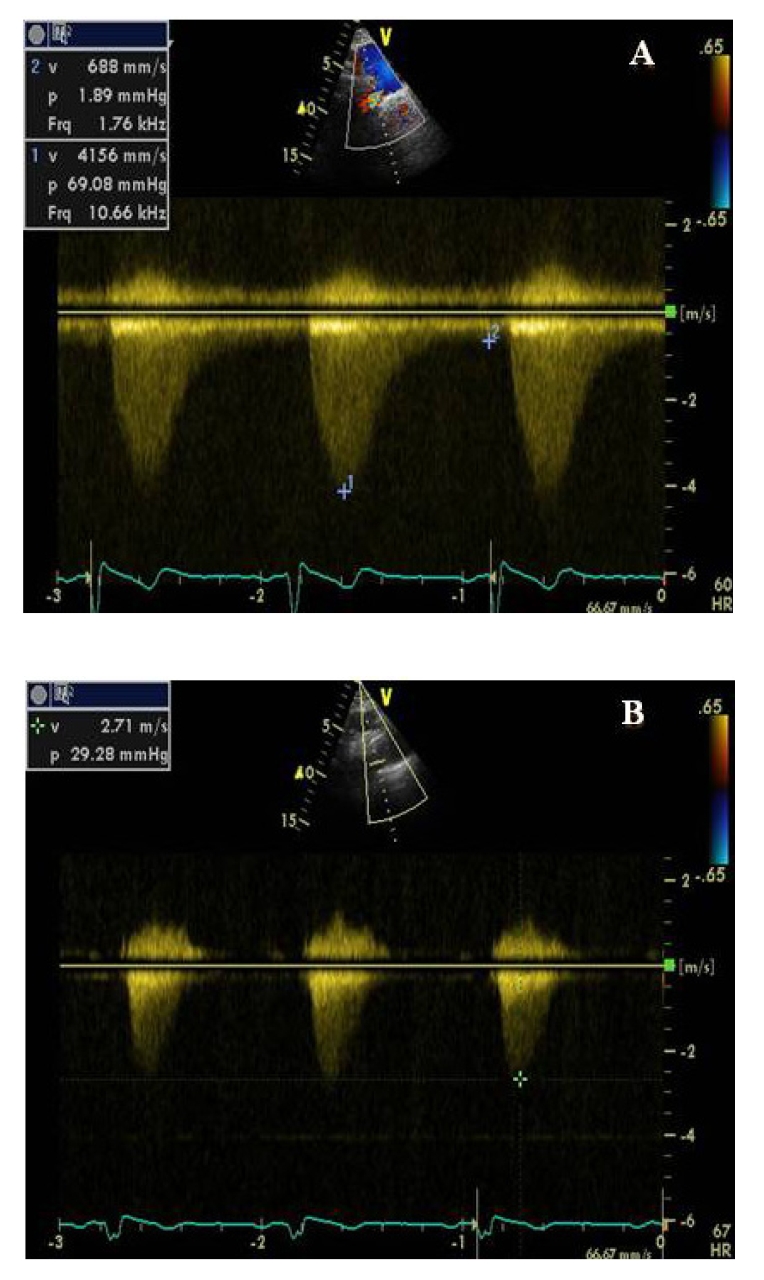
A. Continuous Doppler echocardiography showing a peak systolic gradient of 69 mmHg and a low grade antegrade diastolic flow in the descending thoracic aorta (saw tooth), highly specific for significant aortic obstruction, before stent placement. B. Reduction of the peak systolic gradient at 29 mmHg after stent placement.

At transthoracic echocardiography, which was otherwise unremarkable, from the suprasternal view, we found the signs of an aortic coarctation, with a peak systolic gradient of 69 mmHg at the coarctation site and diastolic run–off at Doppler evaluation; a hypoplastic abdominal aorta was also noted (13/15 mm) (Figure 3).

**Figure 4:**
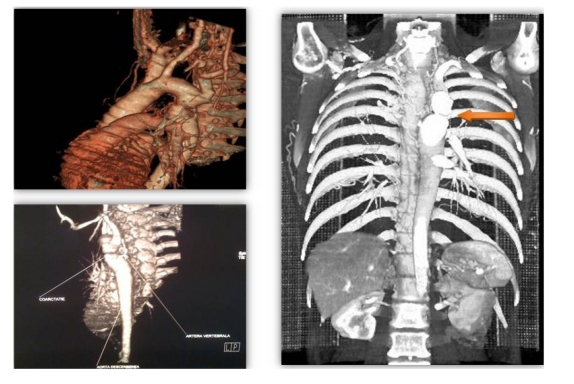
CT 3D images showing significant coarctation of the thoracic aorta beyond the origin of the left subclavian artery (arrow) with poststenotic dilatation and hypoplastic abdominal aorta

For clearly defining the location and anatomy of the coarctation, the patient underwent CT angiography examination that confirmed the presence of the coarctation just distal to the left subclavian artery, with poststenotic dilatation of the descending aorta, with very few collateral vessels (Figure 4) and a hipoplastic abdominal aorta.

**Figure 5:**
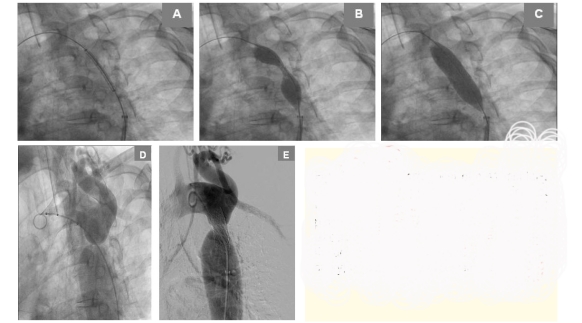
A, B, C. Deployment of a 41 mm long coverd stent mounted on a 16 mm balloon at the coarctation site. The balloon is inflated progressively. D. Aortography before stent placement (showing tight coarctation of the thoracic aorta just beneath the subclavian artery). E. aortography after stent placement.

Considering the presence of high gradient aortic coarctation in the presence of hypertension, corrective treatment was considered mandatory. The treating cardiologist–interventional cardiologist team, matching the patients' preference, chose an interventional method. The patient underwent balloon angioplasty with stent placement. The femoral artery and vein were cannulated, the stenosis was passed with a probe and a guide, the pressure gradient across the coarctation segment was measured and found to be 70 mmHg, afterwards, a long sheat was introduced and a covered stent was positioned at the coarctation site. The stent was a stainless steel open cell stent balloon expandable laser cut 316L with a covering made of expanded PTFE on the interior and exterior aspects, with a 41 mm length and premounted on a 16 mm balloon. Rapid ventricular pacing at a frequency of 170 bpm was performed in order to provide transient decrease in cardiac output and aortic mobility, to achieve balloon stability. When the desired frequency and stability was obtained, the balloon was inflated and the stent fixed on the aortic wall. (Figure 5) The gradient was measured again and it was found to be less than 10 mmHg.

Immediately after the stent placement, clinical reevaluation showed palpable pulses of the dorsalis pedis, posterior tibial, popliteal and femoral arteries and normal blood pressure with minimal treatment (decreased dose of beta blocker and ACE) was achieved during the following days. At echocardiography, from the suprasternal view, the proximal end of the stent that could be seen and the measured peak gradient at this level was of 29 mmHg. 

The patient was discharged three days after the procedure with recommendations for clinical and echocardiographic follow–up at 3 months and yearly afterwards, with an angioCT performed at 5 years intervals. Clopidogrel 75 mg for 3 months and aspirin for 6 months were prescribed. 

At the 6–month follow–up visit, the clinical exam showed preserved lower–limbs pulses, the patient needed low doses of antihypertensive therapy; echocardiography showed a peak systolic gradient in the descending aorta of 27 mmHg. 

## Discussion

Coarctation of the aorta (CoA) is typically a discrete narrowing of the thoracic aorta just distal to the left subclavian artery. However, the constriction may be proximal to the left subclavian artery or rarely in the abdominal aorta. In some cases, coarctation presents as a long segment or a tubular hypoplasia [1]. The most frequently associated lesions include bicuspid aortic valve (up to 85% of the cases), different levels of aortic stenosis, mitral valve stenosis (parachute mitral valve, a complex known as Shone syndrome). Coarctation of the aorta imposes significant afterload on the LV, resulting in increased wall stress, compensatory LVH, LV dysfunction, and the development of arterial collaterals. Aortic coarctation presenting during adult life, most frequently represents cases of re–coarctation, following previous transcatheter or surgical therapy, or missed cases of native coarctation. Aortic coarctation may be recognized in the adult, usually because of systemic arterial hypertension and discrepant upper– and lower–extremity pulses. Patients may complain of exertional headaches, leg fatigue or claudication. 

The reduced life expectancy of patients without correction due to several complications like systemic hypertension accelerated coronary heart disease, stroke, aortic dissection, and congestive heart failure [4], demand an early treatment in these patients.

There are different methods employed for the treatment of CoA in adults, including surgical or percutaneous balloon angioplasty with or without stent placement, and medical therapy.

The 2008 American College of Cardiology/American Heart Association (ACC/AHA) guidelines for adults with congenital heart disease (ACHD), recommend intervention for coarctation in the following circumstances [4]: peak to peak coarctation gradient greater than or equal to 20 mmHg, or peak to peak coarctation gradient less than 20 mmHg, in the presence of anatomic imaging evidence of significant coarctation with radiologic evidence of significant collateral flow (class IC indication). The peak–to–peak gradient is a measurement derived from catheterization data in which the peak pressure beyond the coarctation is subtracted from the peak pressure proximal to the coarctation, and is usually somewhat lower to the peak systolic gradient obtained by echocardiography.

The European Society of Cardiology 2010 guidelines for management of grown–up congenital heart disease say that all patients with a non–invasive pressure difference>20 mmHg between upper and lower limbs, regardless of symptoms but with upper limb hypertension (>140/90 mmHg in adults), pathological blood pressure response during exercise, or significant LVH, should have the intervention (class IC indication) [5].

*Surgical repair* of coarctation can be achieved by several techniques: resection with end–to–end anastomosis, subclavian flap aortoplasty in infants with long–segment coarctation, a bypass graft across the area of coarctation when the distance to be bridged is too long for an end–to–end repair or prosthetic patch aortoplasty [6]. Problems with these techniques have included a significant incidence of aneurysm formation with Dacron patch aortoplasty, and an unacceptably high recoarctation rate with the subclavian flap aortoplasty. The technique of extended end–to–end anastomosis appears to give good short–term to intermediate–term results with a low complication rate and has gained in popularity as the technique of choice when possible to use [7]. A complication associated with all the surgical techniques is aortic dissection, which can occur even late after surgical repair. Surgical mortality is rare (usually less than 1 percent). Morbidity includes early postoperative paradoxical hypertension, left recurrent laryngeal nerve paralysis, phrenic nerve injury, and subclavian steal.  Paraplegia due to spinal cord ischemia and mesenteric arteritis with bowel infarction are rare complications [2]. 

*Balloon angioplasty* is a technique that was first introduced in 1982 and is currently being used either alone or along with stent deployment in the coarcted segment. Balloon angioplasty has been recommended as the preferred treatment for children and adults with native coarctation or recoarctation after surgery [8]. The initial success rate, defined as a gradient < 20 mmHg across the coarctation, is approximately 80 to 90% in the largest studies. The major drawback of angioplasty alone is recoil of the vessel wall with recurrence of stenosis. Balloon angioplasty of the aorta can cause intimal and medial tears resulting in aortic wall dissection in 1–4% of patients, and aneurysm formation in 4–11.5% [1]. Shaddy et al compared the results of angioplasty with surgery in 36 patients [9]. They concluded that the immediate gradient reduction was similar in both modalities. However, there was an increased incidence of aneurysm formation and restenosis after balloon angioplasty. Following balloon dilation, approximately 21x2013;37% of the patients remain hypertensive.

*Stenting of the aortic coarctation* was first introduced in the early 1990s, using bare metal stents. When using this type of stents, the acute mortality rate is 0–3%, whereas neurological complications have not been encountered [2]. Due to the large sheath sizes required, groin hematomas are prevented by the use of perclose and haemostatic devices, although interruption of the femoral and iliac vessels can occur during advancement of the long sheath. Acute aortic dissection and aneurysms following bare stent implantation may be seen in up to 13% of the patients. Stent implantation may theoretically overcome some of the shortcomings of balloon dilatation, because the metal scaffolding may reduce the incidence of acute elastic recoil as well as late restenosis, due to a more complete elimination of gradient in the high–velocity arterial system flow [1]. Reduction or discontinuation of anti–hypertensive therapy following stent implantation is achieved in 41–88% of the patients. 

*Hamdan et al*, reported the results of stent placement in 33/34 patients (13 native and 21 recoarctation) [10]. All but one patient had a successful outcome following stent placement. Two patients underwent successful repeated stent dilation for 16–21 months following the primary stent implantation. Six patients (18%) had complications including two patients who required surgery. The complications were retroperitoneal bleeding secondary to a high puncture (n = 1), ruptured balloon segment embolization to the left axillary artery (n = 1), stent migration (n = 2) requiring additional stent placement, femoral arteriovenous fistula (n = 1), which resolved spontaneously. No patient developed an aneurysm.

*Covered stents* have been extensively used outside the United States in order to address the problems associated with aortic wall injury by balloon angioplasty and ‘bare’ stent placement. Covered stents are preferentially placed in patients where an aortic wall aneurysm exits, where a tight native coarctation is present and balloon or ‘bare’ stent dilatation can be associated with the risk of dissection or rupture; where there is an associated arterial duct; and in older patients in whom the vessel wall is relatively less compliant. The limitations of using a covered stent are the larger sheath size and occlusion of the local branches of the aorta. Occlusion of the left subclavian artery is well tolerated; however, an intact vertebrobasilar system should be documented prior to the procedure [3]. The main risk is related to occlusion of spinal cord arteries, which can result in severe neurological complications like paraplegia. However, the spinal artery usually originates below the level of the ninth thoracic vertebra from the aorta below the diaphragm (in 90% cases), therefore, spinal artery occlusion is unlikely to occur. However, operators should keep in mind that stent migration with fixation of the dislocated stent by redilation in a smaller distal area of the aorta as a rescue procedure might potentially occlude critical side branches that it crosses. The covered stents are balloon–expandable or self–expanding. Also the biodegradable stents are developing [11, 12].

In cases of a very tight coarctation of the aorta, a staged approach may be a safer option. For example, in an ‘hour glass’ deformity, the stent is not fully expanded across the coarctation segment, and, as long as the stent is in a stable position, aggressive dilatation of the stent during initial deployment is not performed, instead, a staged approach is used, with a repeated balloon re–dilatation of the same PTFE covered balloon mounted stent (or of a bear stent) with a repeated dilatation at intervals of 3 to 6 months.

In addition, if a patient remains hypertensive with echo–Doppler findings that show substantial residual gradient, repeated dilatation of the stent is a feasible option. 

The choice of the best coarctation repair method is based on several factors. Stent implantation carries the lowest morbidity whereas repeat interventions are more common following endovascular treatment compared to surgery. In addition, the average duration of hospitalization following transcatheter therapy of aortic CoA is 48 hours, as compared to 5–14 days following surgical relief. Endovascular therapy is currently the treatment of choice when there is ventricular dysfunction or other significant comorbidity, such as diabetes, coronary disease, previous neurological insults, and in older patients with multiple comorbidities [2].

*Medical treatment* of the coarctation of the aorta is focused on hypertension. This should be controlled by beta–blockers, ACE inhibitors, or angiotensin–receptor blockers as first–line medications. The choice of beta–blockers or vasodilators may be influenced in part by the aortic root size, the presence of aortic regurgitation, or both. [4,5]

There are no studies to address the issue of antiplatelet therapy after percutaneous balloon angioplasty with stent placement, but it is considered cautious to recommend double antiplatelet therapy (aspirin and clopidogrel) for 3 months until a complete reendothelialization of the stent.   

Patients with uncomplicated native coarctation or uncomplicated, recurrent coarctation that is successfully repaired, do not require endocarditis prophylaxis unless there is a prior history of endocarditis or a conduit that has been inserted or if surgical repair or stenting has been performed less than 6 months previously.[4]

Survival of patients with aortic coarctation has dramatically improved after surgical repair became available. However, survivors have decreased life expectancy and cardiovascular complications are frequent. Concern falls chiefly in seven categories recoarctation, aortic aneurysm formation or aortic dissection, coexisting bicuspid aortic valve, endocarditis, premature coronary atherosclerosis, cerebrovascular accidents and systemic hypertension.

Furthermore, patients with apparently successful repair of coarctation of the aorta have increased proximal aortic stiffness that might contribute to the known significant cardiovascular mortality and morbidity. The increase in such large artery stiffness reduces the aortic reserve and creates stronger reflected waves, which, themselves, can lead to adverse left ventricular (LV) remodeling and functional disturbances. The effect of stenting on the proximal aortic elastic properties and its relation to cardiac structure and function in such patients was recently studied and abnormal proximal aortic properties with significant evidence of increased arterial wall stiffness before aortic stenting were demonstrated; that is why they remained unchanged afterwards despite the successful relief of downstream obstruction. However, the study showed a significant decrease in the mass index and an improvement in long–axis function of the left ventricle, thus suggesting some degree of reverse remodeling within the few months after removal of the afterload.[13]

Nowadays, it is generally accepted that these patients require indefinite follow–up by a cardiologist, specialized in the field of congenital heart disease. The frequency in which outpatient visits and tests should take place is highly dependent on the clinical history, the presence of associated cardiac anomalies, type of repair, and the patient's blood pressure. [14]

The guidelines [4] recommend that patients who have had surgical repair or percutaneous intervention for coarctation of the aorta should have at least a yearly follow–up and, the evaluation of the coarctation repair site by MRI/CT, should be performed at intervals of 5 years or less, depending on the specific anatomic findings before and after repair (Class I, Level of evidence C). Even if the coarctation repair appears to be satisfactory, late postoperative thoracic aortic imaging should be performed to assess for aortic dilatation or aneurysm formation. Magnetic resonance imaging has a limited role in CoA after stent placement since the metallic artifact (or noise) prevents detailed evaluation of the aortic segment within the stent, despite adequate visualization of the aorta proximal and distal to the stent.

Taking into account that our patient was a 46 year–old lady, with a tight native coarctation of the aorta (gradient of 70 mmHg) and poststenotic dilatation of the descending aorta, the implantation of a covered stent was the best choice in this case because of the high risk of dissection or rupture by using classic stenting. 

## Conclusions

The case presented best illustrates that coarctation of the aorta is a congenital cardiac malformation that can go undiagnosed until old age, having only hypertension as a marker of its presence, because clinical signs can be subtle and overlooked if a complete physical exam is not performed. 

Nowadays, different surgical and interventional types of treatment are available but this should be individualized for each patient and for each type of coarctation (native coarctation or recoarctation after surgical or interventional treatment). 
